# How to spot ocular abnormalities in progressive supranuclear palsy? A practical review

**DOI:** 10.1186/s40035-019-0160-1

**Published:** 2019-07-10

**Authors:** Onanong Phokaewvarangkul, Roongroj Bhidayasiri

**Affiliations:** Chulalongkorn Center of Excellence for Parkinson Disease & Related Disorders, Department of Medicine, Faculty of Medicine, Chulalongkorn University and King Chulalongkorn Memorial Hospital, Thai Red Cross Society, Bangkok, Thailand

**Keywords:** Parkinsonian disorders, Progressive supranuclear palsy, Ocular abnormalities, Early detection, Literature review, Visual observation, Bedside examination

## Abstract

**Background:**

For parkinsonian disorders, progressive supranuclear palsy (PSP) continues to be significant for differential diagnosis. PSP presents a range of ocular abnormalities that have been suggested as optional tools for its early detection, apart from the principal characteristic of postural unsteadiness. Nonetheless, such symptoms may be difficult to identify, particularly during the early onset stage of the disorder. It may also be problematic to recognize these symptoms for general practitioners who lack the required experience or physicians who are not specifically educated and proficient in ophthalmology or neurology.

**Main body:**

Thus, here, a methodical evaluation was carried out to identify seven oculomotor abnormalities occurring in PSP, comprising square wave jerks, the speed and range of saccades (slow saccades and vertical supranuclear gaze palsy), ‘round the houses’ sign, decreased blink rate, blepharospasm, and apraxia of eyelid opening. Inspections were conducted using direct visual observation. An approach to distinguish these signs during a bedside examination was also established. When presenting in a patient with parkinsonism or dementia, the existence of such ocular abnormalities could increase the risk of PSP. For the distinction between PSP and other parkinsonian disorders, these signs hold significant value for physicians.

**Conclusion:**

The authors urge all concerned physicians to check for such abnormalities with the naked eye in patients with parkinsonism. This method has advantages, including ease of application, reduced time-consumption, and requirement of minimal resources. It will also help physicians to conduct efficient diagnoses since many patients with PSP could intially present with ocular symptoms in busy outpatient clinics.

**Electronic supplementary material:**

The online version of this article (10.1186/s40035-019-0160-1) contains supplementary material, which is available to authorized users.

## Background

Progressive supranuclear palsy (PSP, Steele-Richardson-Olszewski syndrome) is a neurodegenerative parkinsonian disorder, classically described as a syndrome of postural instability, supranuclear vertical gaze palsy, dysarthria, dystonic rigidity of the neck and trunk, mild dementia, and pseudobulbar palsy [1]. It represents an important differential diagnosis of Parkinson’s disease (PD), accounting for approximately 6% of all cases of degenerative parkinsonism referred to specialist clinics [2]. The National Institute of Neurological Disorders and Stroke (NINDS) developed a set of clinical research criteria of the syndrome that emphasize the presence of vertical supranuclear gaze palsy or slow vertical saccades as well as early falls due to postural instability as the most useful physical signs that distinguish PSP from PD [3, 4]. Nevertheless, the rate of misdiagnosis of PSP remains high, with up to 50% of cases in routine clinical practice not recognized, partly due to the absence of falls and gaze palsy in many patients [4, 5]. To improve sensitivity toward the different variants of PSP, the new MDS clinical diagnostic criteria of PSP, in combination with specific combinations of certain features, may help optimize early clinical diagnosis of PSP [6].

Supranuclear vertical gaze palsy has been recognized as one of the cardinal features of PSP. However, a clinicopathological study that involved a comprehensive review of case notes of family doctors and medical specialists as well as in-patient notes and consultations indicated that in more than half of the cases early in the disease process, no detailed examination of the eye signs was recorded, beyond a statement of ‘cranial nerve examination normal’ [5]. This may be due to a number of reasons, including a lack of awareness of useful eye findings as part of the diagnostic work-up among treating physicians as well as a lack of eye complaints from patients, resulting in incomplete physical examination. In addition, many general physicians may feel that eye examinations are irrelevant to them and not part of their routine examination, as they consider them to fall under the expertise of eye specialists who try to identify subtle findings.

The early identification of patients with PSP has several benefits to both patients and caregivers, including early referral to specialists, focus treatments being tailored to specific symptoms that impair the patients’ quality of life, and appropriate counselling. Moreover, careful examinations are likely to prevent unnecessary investigations of patients. Eye examinations, particularly eye movement tests, should thus be performed as part of the general examination in patients who primarily present with akinetic-rigid syndromes [7]. Therefore, in this article, we present a systematic review of the existing evidence in the literature on common eye signs in PSP, and propose practical bedside methods on how to elicit them. In many instances, screening eye examinations in patients with parkinsonism are not difficult to perform by general physicians if they know what to look for, and do not take much of the consulting time. Appropriate referrals to specialists, including movement disorder neurologists and ophthalmologists, for specific examinations and management can then be considered in suspected cases of PSP.

## Main text

### Progressive supranuclear palsy for general physicians

As the name entails, PSP is an adult-onset progressive neurodegenerative disorder associated with supranuclear gaze palsy. It is diagnosed in approximately 6% of all parkinsonian patients being referred to a specialized clinic [2]. In the classification of parkinsonism, PSP falls under the category of multiple system degenerations or ‘parkinsonism-plus syndromes’, with the ‘plus’ feature being oculomotor dysfunction, postural disability, akinesia, and cognitive dysfunction [6, 8]. PSP occurs more often in men, with a mean onset age of 63 years, 10 years later than the typical onset of PD [9]. It has distinct neuropathological features associated with tau pathology at different levels of the central nervous system (CNS), leading to an akinetic-rigid syndrome with oculomotor dysfunction, postural instability, and frontal lobe and bulbar dysfunction. Despite the name, supranuclear gaze palsy is not always present, and clinical manifestations, particularly in the early stage, can be variable and subtle, thus making the diagnosis difficult if certain physical signs (i.e. eye signs) are not specifically looked for. Mobility problems, described as recurrent falls, are a common early feature, and visual symptoms, including diplopia, photophobia, and eyelid apraxia are often functionally disabling (see Additional file 1) [10]. In simple terms, the diagnosis of PSP should be considered in all patients presenting with parkinsonism not responding to levodopa, postural instability with falls, executive dysfunction, dysarthria, and, importantly, eye movement abnormalities.

In order to diagnose PSP clinically, the National Institute of Neurological Disorders and Stroke and Society for Progressive Supranuclear Palsy (NINDS-SPSP) has proposed probable and possible clinical diagnostic criteria that have been shown to provide high sensitivity and high positive predictive values suitable for routine clinical care [3, 11]. The NINDS-SPSP criteria rely on the identification of a progressive disorder with onset age after 40 years with a combination of two cardinal features, namely postural instability with falls during the first year of the disease and slow vertical saccades or supranuclear gaze palsy [3]. However, the validation of these criteria in an independent set of patients has demonstrated low sensitivity in patients in the early stage of the disease and in patients presenting with variant PSP syndromes that differ from the classical form of PSP [3, 12, 13].

The classic form of PSP is also called Richardson PSP phenotype (PSP-RS) (also known as Steele-Richardson-Olszewski syndrome); patients report early difficulties with vertical gaze and pseudobulbar palsy, nuchal dystonia, and dementia [14]. Patients with PSP with non-classical presentations have been reported, which account for up to 76% of autopsy-confirmed PSP populations [15]. For non-classical variants (PSP-ocular motor dysfunction, PSP-postural instability, PSP-parkinsonism, PSP-frontal, PSP-progressive gait freezing, PSP-corticobasal syndrome, PSP-primary lateral sclerosis, PSP-cerebellar, and PSP-speech/language disorders), clinical manifestations are much more diverse, with supranuclear gaze paresis developing late in the majority of cases (Table 1) [6, 16]. PSP-parkinsonism represents the most common form among these variants (32%), characterized by an asymmetric onset, tremor, and a moderate initial therapeutic response to levodopa, and is frequently confused with PD [5]. Patients with these variants may not be diagnosable using the current clinical diagnostic criteria [16]. The progression of PSP is more rapid and severe than that of PD, given the variability described among the different clinical subtypes. PSP has a median survival rate of 7.4 years, with the mode of death commonly related to respiratory compromise or falls [9]. Predictors of progression include Richardson syndrome, male gender, and disease onset in older age [17].

**Table 1:** The presence of ocular abnormalities according to chronological order from disease durations including early stage (< 4 years), middle stage (4–8 years), and late stage (> 8 years) among autopsy-proven cases of PSP and its variants

PSP subtypes	SWJ	SS	VSP	RTH	DBR	BSP	ALO
PSP-RS	+++ (early to middle stage manifestation)	++ (early to middle stage manifestation)	+++ (early to middle stage manifestation)	++ (early stage manifestation)	+ (middle to late stage manifestation)	+ (middle to late stage manifestation)	+ (middle to late stage manifestation)
PSP-OM	N/A	N/A	++	N/A	N/A	N/A	N/A
PSP-PI	Absent	+ (late stage manifestation)	+ (late stage manifestation)	Absent	Absent	Absent	+ (late stage manifestation)
PSP-P	N/A	+ (middle to late stage manifestation)	+ (middle to late stage manifestation)	N/A	N/A	N/A	N/A
PSP-Frontal	Absent	Absent	Absent	Absent	Absent	Absent	Absent
PSP-PGF	Absent	+ (middle to late stage manifestation)	+ (middle to late stage manifestation)	Absent	Absent	+ (late stage manifestation)	Absent
PSP-CBS	N/A	+ (early to middle stage manifestation)	+ (early to middle stage manifestation)	N/A	N/A	N/A	N/A
PSP-PLS	Absent	Absent	Absent (still absent over 10-year duration)	Absent	Absent	Absent	Absent
PSP-C	N/A	N/A	+ (early to middle stage manifestation)	N/A	N/A	N/A	N/A
PSP-SL	Absent	Absent	+ (middle stage manifestation)	Absent	Absent	Absent	Absent

The information described in this table was pooled from this systematic review

*SWJ* Square wave jerks, *SS* Slow saccades, *VSP* Vertical supranuclear palsy, *RTH* Round the house sign, *DBR* Decreased blink rate, *BSP* Blepharospasm, *ALO* Apraxia of eyelid opening, *PSP-OM* PSP-ocular motor dysfunction, *PSP-PI* PSP-postural instability, *PSP-P* PSP-parkinsonism, *PSP-frontal* PSP-frontal, *PSP-PGF* PSP-progressive gait freezing, *PSP-CBS* PSP-corticobasal syndrome, *PSP-PLS* PSP-primary lateral sclerosis, *PSP-C* PSP-cerebellum, and *PSP-SL* PSP-speech/language disorders

One of the main reasons why the diagnosis of PSP is delayed by several years after the disease onset is that the oculomotor cardinal features (slow vertical saccades and supranuclear gaze palsy) may develop late or never during the course of the disease [6, 15, 18]. A list of differential diagnosis is presented in Table 2. Consequently, the new MDS clinical diagnostic criteria of PSP have been proposed and stratified by three degrees of diagnostic certainty (probable PSP, possible PSP, and suggestive PSP). These criteria are related to the four functional domains where characteristic clinical manifestations of PSP appear, including ocular motor dysfunction, postural instability, akinesia, and cognitive dysfunction [6]. Based on the current available evidence, these criteria provide an optimized early, sensitive, and specific clinical diagnosis of PSP [6].

**Table 2:** Differential diagnosis of supranuclear gaze palsy and parkinsonism

Aetiology	Disorders
• Neurodegenerative	• Progressive supranuclear palsy (PSP)
	• Dementia with Lewy body (DLB)
	• Corticobasal degeneration (CBD)
	• Frontotemporal dementia (FTD)
	• Alzheimer’s disease (AD)
• Heredodegenerative	• Kufor Rakeb disease
	• Niemann-Pick disease, type C
	• Perry syndrome
	• Mitochondrial disease (POLG)
	• Dentatorubral pallidoluysian atrophy
	• Gaucher disease
	• Huntington’s disease
	• Wilson’s disease
	• Neuroacanthocytosis
• Vascular	• Vascular-Progressive supranuclear palsy (Vascular-PSP)
	• Cerebral autosomal dominant arteriopathy with subcortical infarcts and leukoencephalopathy (CADASIL)
• Infection	• Neurosyphilis
	• Whipple’s disease
• Prion	• Prion’s disease
• Immune-mediated	• Paraneoplastic encephalitis (Anti-Ma1, Anti-Ma2 antibodies)

**Fig. 1:**
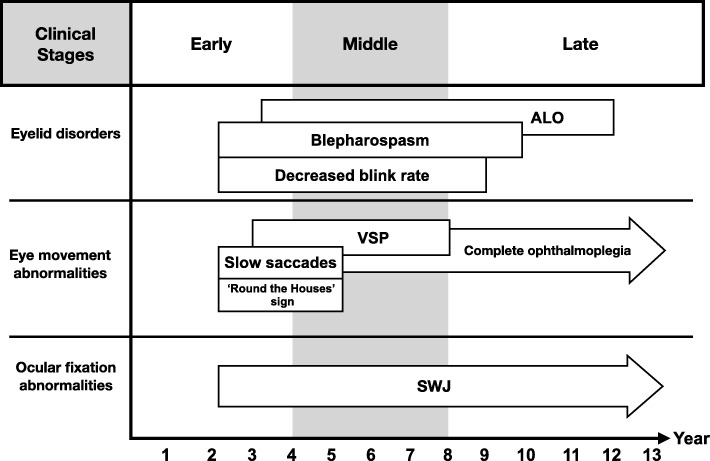
Evolution of ocular abnormalities in PSP presented in chronological order. The clinical stages are divided into three stages related to clinical appearance, namely (1) early stage: clinical features apparent in less than 4 years of disease duration; (2) middle stage: clinical feature apparent for between 4 and 8 years of disease duration; and (3) late stage: clinical feature apparent for more than 8 years of disease duration. Solid white rectangles indicate timeline occurrences of each ocular abnormality, presented in years after onset of disease. Solid arrows indicate the presence ophthalmoplegia and square wave jerks, which continue to be present in the late disease stage

Based on distinctive neuropathological features, PSP can be divided into several clinical subtypes, providing some guidance on the prognosis and natural history [5, 6, 16]. Patients with the classic form of PSP (PSP-RS) usually report early difficulties with balance, personality changes, and visual disturbances in association with initial square-wave jerks, slow vertical saccades evolving into hypometric saccades, supranuclear vertical gaze palsy, and eventually eyelid abnormalities (Fig. 1) [3, 4, 19]. From this systematic review, we are able to identify the occurrence of ocular abnormalities in chronological order as shown in Fig. 1. While their presence may evolve in the given fashion, this is not the rule and variations may indeed occur.

### Neuropathological correlations of oculomotor abnormalities in PSP

**Fig. 2:**
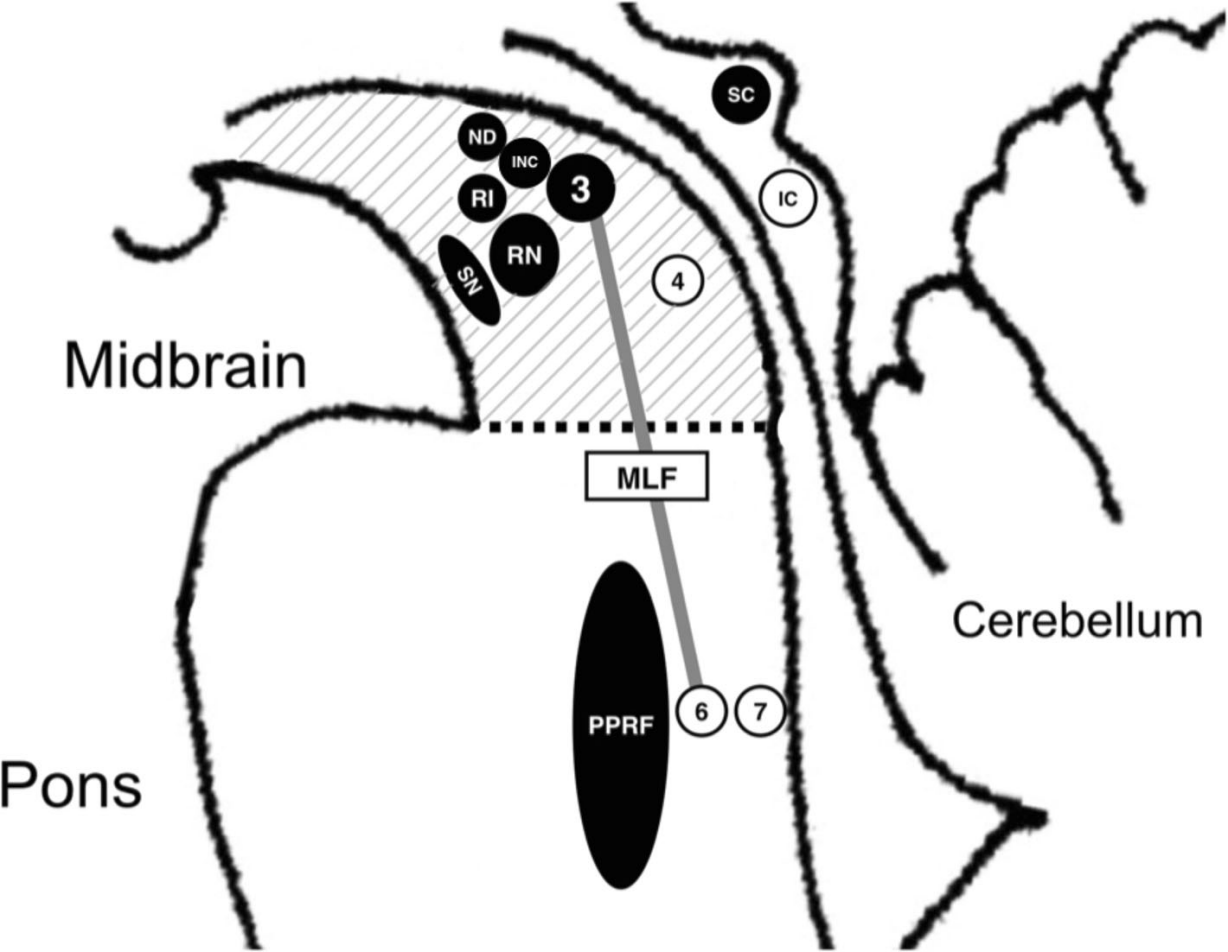
Sagittal section of human brainstem showing the locations of the degeneration of brainstem nuclei responsible for the presence of ocular abnormalities in PSP. Black circles represent the degeneration of some specific brainstem structures resulting in ocular abnormalities. Abbreviations: riMLF: rostral interstitial nucleus of the medial longitudinal fasciculus, RN: red nucleus, INC: interstitial nucleus of Cajal, PPRF: paramedian pontine reticular formation, MLF: medial longitudinal fasciculus, SC: superior colliculus, IC: inferior colliculus, 3: nucleus of the oculomotor nerve, 4: nucleus of the trochlear nerve, 6: nucleus of the abducens nerve, 7: nucleus of the facial nerve

The constellation of parkinsonism and cognitive, speech, and oculomotor features in PSP reflect marked neuronal degeneration at different levels of the CNS, including the basal nucleus of Meynert, the pallidum, subthalamic nucleus, superior colliculi, mesencephalic tegmentum, substantia nigra (both pars compacta and reticulata), locus coeruleus, red nucleus, reticular formation, vestibular nuclei, cerebellum, and spinal cord (Fig. 2) [14, 20, 21].

In PSP, two oculomotor defects are prominent, namely, a restricted range of vertical movement and vertical saccadic slowing [19]. Slow vertical saccades, a cardinal early feature of PSP, reflect abnormalities in the brainstem neural network affecting primarily burst neurons in the rostral interstitial nucleus of the medial longitudinal fasciculus (riMLF) [22, 23]. As the interstitial nucleus of Cajal (INC) contributes to vertical gaze holding, inactivation of this structure causes a restricted range of vertical eye movement without inducing slow saccades [24]. As the disease progresses, slowing of horizontal saccades is also evident, suggesting the involvement of burst neurons in the parapontine reticular formation (PPRF) as well [23, 24]. These structures are located in the mesencephalic reticular formation, which is involved in PSP. Table 3 summarizes the correlations between neural substrates and oculomotor signs in PSP.

**Table 3:** The common types of ocular abnormalities, their clinical findings, and pathological correlation

Eye examination	Clinical findings	Anatomical correlation of degeneration
Ocular fixation	Square wave jerks	Paramedian pontine reticular formation (PPRF)
Eye movement	Slow saccades (vertical)	Rostral interstitial nucleus of medial longitudinal fasciculus (riMLF)
	Vertical supranuclear gaze palsy	Interstitial nucleus of Cajal (INC)
		Substantia nigra par reticulata
		Superior colliculus
	‘Round the Houses’ sign	Inconclusive data
Eyelids	Decreased blink rate	Inconclusive data
	Blepharospasm	Inconclusive data
	Apraxia of eyelid opening	Inconclusive data

### Different types of oculomotor abnormalities in progressive supranuclear palsy: a systematic review

To determine different types of oculomotor abnormalities that were reported in patients with PSP, we conducted a systematic review by performing an electronic database search on PubMed, EMBASE, Cochrane Library, and life science journals. The following terms were used for the literature search: *Progressive supranuclear palsy* AND *gaze* OR *gaze palsy* OR *saccades* OR *slow saccades* OR *fixation* OR *saccadic intrusion* OR *square wave jerks* OR *round the houses sign* OR *apraxia of eyelid opening* OR *blepharospasm* OR *blink rate* OR *eye blink* OR *decreased blink rate* OR *eye* OR *eyelid* OR *ocular* OR *visual* OR *vision* OR *signs* OR *symptoms* OR *abnormalities* OR *impairment* OR *disorders*. A target search of bibliographies of relevant articles was also performed to identify additional articles for inclusion. Titles and abstracts were identified from database searches and chosen according to the aforementioned selection criteria. Additional abstracts were identified from manual searches (authors’ libraries and additional papers quoted in the retrieved papers). Only original full-text articles on PSP related to searching items published in English between January 1973 and December 2018 were included in this review. Reviews and editorial articles presenting no original data were excluded. Both assessors (OP, RB) independently screened each paper and were required to agree on each study in order to be included in this review. We screened 2745 titles and abstracts, from which 117 full-length articles were selected for further review. Of these, 45 articles fulfilled the selection criteria (see Additional file 2). Findings on oculomotor abnormalities were categorized into disorders of ocular fixation, eye movements, and eyelids. Seven oculomotor abnormalities, including square wave jerks, speed and range of saccades (slow saccades and vertical supranuclear gaze palsy), ‘round the houses’ sign, decreased blink rate, blepharospasm, and apraxia of eyelid opening, were selected based on their prevalence, distinctive features, and simplicity to elicit during a bedside examination. To take into account clinical relevance, we structured the presentation of each abnormality according to its definition, examination techniques with supplemented video clips (see Additional files 3 and 4), and clinical implications.


**Additional file 3:** Video of the common seven eye signs of patients with progressive supranuclear palsy ordered as following; square wave jerks, slow saccades, ‘round the houses’ sign, vertical supranuclear gaze palsy, decreased blink rate, blepharospasm, and apraxia of eyelid opening. (MP4 14427 kb)


### Square wave jerks

#### What are square wave jerks?

Square wave jerks (SWJs) represent the most common type of saccadic intrusions, characterized by conjugated couplets of horizontal back-to-back microsaccades (0.5–5°), taking the eye from the fixation point and back onto it in an involuntary manner, with a normal intersaccadic interval of about 200 ms [25]. Saccadic intrusions are involuntary conjugate saccades that interrupt fixation, and can be sub-classified by the presence (SWJs, square wave pulses, and macrosaccadic oscillations) or absence (ocular flutter and opsoclonus) of an intersaccadic interval [25].

#### How to elicit square wave jerks

SWJs can be identified by asking the patients to maintain their gaze fixation to a static object for the duration of 10 to 30 s. Alternatively, SWJs can be observed with Frenzel’s goggles or an ophthalmoscope, by asking the patients either to hold their gaze steadily at the primary position or to shift their gaze towards eccentric positions. On clinical inspection, SWJs are visualized as saccade pairs that appear purely horizontal: the first saccade moves the eye away from the fixation target, and, after a short interval, the second saccade brings it back to the target [26]. In addition to SWJs, microsaccades may be observed, but they are likely to be too small (< 0.5°) to be evident during clinical examinations in normal subjects [27]. While SWJs appear to be purely horizontal on clinical inspection, microsaccades often display an oblique trajectory [28]. Recent studies suggested that both microsaccades and SWJs are generated by the same neural circuit, and that larger saccades are more likely to show SWJs [26, 29].

#### Clinical implications of square wave jerks

It is important to understand that occasional SWJs can occur in preschool children and healthy elderly subjects [30, 31]. The typical frequency of SWJs is less than 9/min in healthy subjects, but the frequency seems to increase with age [32]. However, certain observations, such as large (> 5°), frequent (> 16 per minute during fixation or > 20 per minute in the dark), multiplanar, or disconjugate SWJs, should alert physicians of an underlying neurological dysfunction [33]. While SWJs have been reported in patients with PSP, they are not specific to this disorder, and have been observed in a number of disorders affecting the functions of brainstem circuits, particularly the cerebellum and its output through the superior cerebellar peduncle. However, certain characteristics of SWJs such as macro SWJs were included in the core clinical features suggestive of SPS from the new MDS clinical diagnostic criteria of PSP, which may be useful in the differentiation of individuals with PSP from normal subjects or those with other parkinsonian disorders [6]. These disorders include Friedreich’s ataxia, Huntington’s disease, oculomotor apraxia type 2, PD, multiple system atrophy (MSA), corticobasal degeneration (CBD), and recessive spinocerebellar ataxias [34, 35].

Out of 45 articles included in this systematic review, nine articles studied the presence of SWJs in patients with PSP using different eye recording techniques [26, 34, 36–42].

Among various forms of parkinsonian syndromes, SWJs were reported in most patients with PSP (60–100%), followed by MSA (40–60%), CBD (33%), and, to a lesser extent), PD (0–15% of patients) [34, 36, 38]. The frequency of SWJs reported in the literature may vary depending on the respective recording technique. Moreover, SWJs in patients with PSP were identified to be larger than 1°, which is a useful clinical sign for differentiation of PSP from PD, since large SWJs or macro SWJs are considered uncommon among patients with PD [34]. When compared to healthy normal subjects, SWJs in patients with PSP were more frequent, larger, and more markedly horizontal [36, 41]. Microsaccades also lost their vertical component in patients with PSP [26]. The latter finding has been identified as the most distinctive characteristic sign of PSP [26].

Other studies looked further into fixational characteristics among patients with parkinsonian disorders [36, 39, 42]. A high proportion of abnormal ocular fixations was observed in patients with PSP, MSA, or CBD [34, 38]. Patients with PSP exhibited larger SWJs than controls when fixation targets were visible, and this difference was reversed in the absence of a fixation target [39, 40]. In contrast, patients with MSA demonstrated increased SWJs in conditions with and without a visible target [36]. Whether this observation represents a useful tool to discriminate PSP from other parkinsonian disorders in the early stage is still unclear, and these findings need to be replicated in subsequent studies. Another study described the distinguished patterns of SWJs in PSP, which showed larger sizes, higher frequencies, but less vertical components than SWJs in control subjects. The authors noted that this finding is consistent with the typical feature of PSP, vertical supranuclear palsy, and suggested that SWJs should be considered an early eye sign in PSP, due to the short disease duration and early symptom onset (median duration of 4 years) [26].

SWJs can result from either (1) disruption to areas that influence saccadic control, including the cortex, cerebellum, superior colliculus, and basal ganglia, or (2) direct injury to omnipause neurons located in the brainstem [37]. One study presented similar explanations for SWJ mechanisms, and proposed the hypothesis that the abnormality in supranuclear control might interrupt omnipause cell activity in paramedian pontine reticular formation, resulting in the release of saccadic burst units [43]. Another proposed mechanism for SWJs might be reflected in the degeneration involving the cerebellum and its connections, such as to the superior cerebellar peduncle, and result in abnormal to-and-fro eye movements during attempted steady fixation [19, 44]. This hypothesis is supported by the proposed relevance of SWJs in the impairment of visual scanning during attempt fixation among patients with cerebellar ataxia [45, 46].

### Slow saccades and vertical supranuclear gaze palsy

#### What are slow saccades and what is vertical supranuclear gaze palsy?

Saccades are rapid eye movements that redirect the foveal line of sight toward features of interest so that they can be seen optimally [25]. The speed and range of saccades represent important components of saccadic examination, and their abnormalities reflect the dysfunction of the burst neurons (riMLF for vertical saccades and PPRF for horizontal saccades) that are frequently affected by the disease process in PSP [22, 23]. Since saccades are the fastest eye movements (up to 500°/s) with very brief duration (< 100 ms), the examiner should not expect to be able follow the full trajectory of normal saccades with their own eye [25]. The normal duration of a saccade is 30–100 ms for amplitudes ranging from 0.5 to 40° [47]. The larger the saccade, the higher the peal velocity. Saccades are slower when made in darkness to a remembered target location and when made in the opposite direction to a visual stimulus (the ‘antisaccade test’) [48].

In general terms, gaze palsy refers to deficits in conjugate eye movements that affect both eyes. Therefore, gaze palsy, when present, is usually indicative of supranuclear lesions resulting from interruption of the neural pathways that carry commands for voluntary saccades and pursuit before they reach the eye movement ‘generators’ in the brainstem. Dysfunction of the generators, namely the riMLF, results in vertical supranuclear gaze palsy, while dysfunction of the PPRF can lead to horizontal gaze palsy [22].

#### How to test saccadic velocity and gaze palsy

Testing for the velocity of saccades can be performed when the subject is asked to glance back and forth between two horizontal and vertical targets. Not only the velocity, but also the accuracy and conjugacy of the saccades should be observed. Normal individuals can reach the target with a single fast movement or with one small corrective saccade. It is important for the examiner to ensure that the subjects pay full attention during the performance of saccades, since patients who are drowsy, inattentive, or heavily medicated may produce normal saccades with slow velocity [49]. Moreover, there is a very strong age effect in the performance of saccades with a tendency for elderly subjects (60 years and over) to produce slower and delayed saccades, compared to younger individuals [50].

In order to determine slow saccades, researchers have utilized different quantitative methods of eye movement recordings, ranging from electrooculography, scleral search coil systems, and video-oculography. However, the identification of slow saccades on clinical examination is more challenging, particularly in patients with mildly slow saccades. If the examiner can follow the full trajectory of the patient’s saccades with their own eye, the saccades of that particular patient are likely to be slow [51]. Another useful clinical pearl is that patients with delayed saccadic initiation and slow saccades often blink or orient their head towards the target before making a saccade [52]. Different methods have been proposed to enhance the recognition of slow saccades, including the use of an optokinetic drum, or the use of oblique targets that will result in an L-shape saccade [52]. However, these methods have not been tested in patients with PSP to demonstrate their sensitivity and specificity. In suspected cases of PSP, particular attention should be paid to the velocity of vertical and horizontal saccades independently, since selective slow vertical saccades are observed in the early stage, followed by slow horizontal saccades when the disease is more advanced [23].

The range of motion of both horizontal and vertical saccades can be tested in a self-paced or verbally guided manner. Before determining that gaze palsy is pathological, it is important for physicians to be aware that a limitation of the vertical range of eye movements, especially upgaze, can occur in elderly subjects; the velocity should however not be affected in such cases [53]. In addition to gaze palsy, features suggesting supranuclear lesions should be specifically looked for. This can be accomplished by assessing improvements in the gaze palsy with a vestibuloocular reflex, thereby bypassing volitional pathways.

#### Clinical implications of slow saccades and vertical supranuclear gaze palsy

Out of 45 articles included in this systematic review, 10 articles studied slow saccades in patients with PSP [10, 23, 37, 38, 40, 41, 54–57], and 19 articles studied VSP in patients with PSP [1, 4, 10, 15, 18, 19, 36, 37, 41, 54, 58–66], identified using different eye recording techniques.

In the setting of parkinsonian syndromes, the identification of VSP is highly specific for the diagnosis of PSP, with the exception of a few case reports of dementia with Lewy bodies and CBD [67–70]. While VSP constitutes one of the mandatory inclusion criteria for the clinical diagnosis of probable PSP, proposed by the NIND-SPSP [3], a real clinical problem is that not all patients with PSP exhibit the full range of eye movement abnormalities, particularly in the early stage [37, 42, 55, 56]. VSP should, however, not be used as an early sign in PSP, as it usually presents in the advanced disease course (range of follow-up periods: 1–10 years) [5, 19, 54, 60–62, 66, 71]. In addition, some patients with PSP, especially those with variants who meet the clinic-pathologic criteria, might be diagnosed based on the pathological confirmation of the diagnosis without existing VSP or in spite of a lack of other eye movement abnormalities [4, 5, 59, 60, 63, 72–75].

Therefore, the typically slow saccades with curved trajectories are giving a strong indication when observed in patients with early onset parkinsonism, but vertical saccades are more affected than horizontal saccades in PSP [10, 23, 38, 40, 54]. Additionally, both horizontal and vertical saccades are slow and have irregular trajectories and velocity profiles, but deficits have been found to be particularly severe in the vertical axis [57]. A number of clinicopathological studies have been able to correlate the presence of midbrain atrophy with the degeneration of burst neurons in the riMLF, clinically manifested as selective slow vertical saccades rather than horizontal saccades [22, 23]. Several studies of pathologically proven cases of PSP reported a range of 40–90% of patients that were documented to have vertical gaze palsy, implying that vertical gaze palsy was not evident in all patients [4, 5, 15, 58–60, 66, 71, 76–80]. For example, some variants of PSP including PSP-frontal, PSP-PLS, and PSP-SL show no eye movement abnormalities during standard clinical examination at an early stage of the disease [80–82]. In an autopsy-proven case of PSP-PLS, vertical gaze palsy was absent throughout the 10-year disease course [81].

These pathologically studied cases provide strong evidence that, although vertical supranuclear palsy is relatively specific to PSP in the setting of parkinsonian syndromes, it is not a universal finding in this disorder. Additional involvement of cerebellar signs has been proposed for a new subtype of PSP called ‘PSP-cerebellum’ (PSP-C) [63, 66]. In this group of patients, eye signs can be under-recognized during the first 2 years [63, 66]. The real question to clinicians is what types of oculomotor abnormalities can help increase the diagnostic acumen for different variants and stages of PSP. The typical VSP in PSP can usually be partially improved with vestibule-ocular reflex (VOR) manoeuvres, which show an abnormal reciprocal inhibition of antagonist vertical muscles during attempt voluntary movements, while the reciprocal innervation is well preserved during reflex movements [64]. The supranuclear degeneration of the pathway from the substantia nigra pars reticulata to the superior colliculus may be an important cause of gaze palsy in PSP [83]. Our systematic review provides more evidence that slow saccades and vertical supranuclear palsy do not occur at once in patients with PSP. Rather, they tend to evolve as the disease progresses. One longitudinal study of oculomotor function in patients with PSP suggested that slow saccades occur throughout the disease course with preserved saccadic latency [84].

### ‘Round the houses’ sign

#### What is a ‘round the houses’ sign?

Indicating an incapability to make pure vertical saccades along a straight line in the midline and lateral arc, which creates a curve course of oblique saccades, the ‘round the houses’ sign for PSP was mentioned for the first time by Quinn in 1996 [85]. Using the analogy that each eye makes a round excursion in its own ‘house’ (orbit), the sign employs the plural ‘houses’. In addition to midbrain atrophy, the degeneration of the burst neurons in the riMLF likely explains the mechanism of early selective slow vertical saccades rather than horizontal saccades, and may also explain the occurrence of the ‘round the houses’ sign [23]. Furthermore, the paramedian pontine reticular construction is mainly responsible for creating horizontal saccades [22, 85, 86].

#### How is a ‘round the houses’ sign provoked?

By prompting a patient to carry out quick eye movements to a moving target between a completely upright and downwards position while keeping the head stationary, a ‘round the houses’ sign can be detected. Eye motion following a curved course (oblique saccades), rather than a straight line, could also be an indication for a ‘round the houses’ sign [85, 86].

#### Clinical implications of a ‘round the houses’ sign

Two articles (one commentary and one case report) reporting on ‘round the houses’ signs in patients with PSP were identified [85, 86]. The signs were mentioned by Quinn in an old comment through his experience with several patients with PSP [85]. The curved course of oblique saccades might be a result of the viewer conducting vertical upward and downward saccades, resulting in an oval-like trajectory by inconsistent reduction of the vertical saccades compared to the horizontal saccades, evenly full gaze existed [85]. A ‘round the houses’ sign has thus been defined as an early eye sign to look for in patients with PSP [85]. It may also occur together with other signs as a late indicator in patients with advanced PSP, unless it results from vertical supranuclear gaze palsy [85]. The sign was recently described in Niemann-Pick disease type C (NPC) [87, 88], while dystonia, hyperreflexia, and hepatosplenomegaly are the usual indicators of adult-onset NPC [87].

### Decreased blink rate

#### What is a decreased blink rate?

By eliminating irritants from the surface of the cornea and conjunctiva as well as aiding to spread tears covering the globe surface to sustain a normally moist state, blinking and secretion of tears are basic protective mechanisms of the eyes [89]. Consisting of a swift lowering of an upper eyelid mediated by a short contraction of the orbicularis oculi (OO) muscle, eye blinking is linked to the reciprocal inactivation of the levator palpebrae superioris (LPS) muscle of the upper eyelid [89, 90].

#### How is a decreased blink rate assessed?

Bilateral paroxysmal closing of the eyelids (duration: less than 1 s) is the definition of a blink that occurs without being triggered by an outside stimulus. By counting how many times a person blinks per minute during a 5-min conversation, a decreased eye blink rate can be identified [91]. Otherwise, digitally-recorded video images concentrated on the eyes may be utilized. When at least 50% closure of the eye happens during a quick closing and subsequent reopening of the eye, a blink is classified as a ‘full blink’. As prolonged eye closure is typically an intentional act in response to deep thought, it is usually excluded from assessments of blinks. Spontaneous blinks with under 50% of eyelid closure are not considered for the total blink rate [92].

#### Clinical implications of a decreased blink rate

Our methodical analysis showed that four studies found a noteworthy reduction in blink rates among patients with PSP in comparison to healthy controls in terms of unprompted as well as trained blink responses [91, 93–95]. Additionally, patients with PSP exhibited much longer periods of closed eyes during unprompted blinking compared to the healthy controls [95]. Further, patients with PSP showed a significant reduction in their conditioned eye blink response and more verbal memory impairment, resulting in the proposal of a dissociated pattern of learning abilities. In a serial reaction time task, meanwhile, compared to controls, obvious sequence patterns were comparatively sustained [93]. A neurophysiological device employed to gauge brainstem excitability could be advantageous for separating patients with PSP from others with CBS, as found in a study on the benefits of the R2 blink reflex recovery cycle (R2BRRC) [96]. In this study, patients with PSP showed earlier employment of R2BRRC than patients with CBS [96].

Striatal and mesolimbic dopaminergic performance can be assessed via blinking rates. Alterations in rates have been found in various neuropsychiatric disorders, and are thought to come from irregular central dopaminergic functions [97]. Patients with schizophrenia, Tardive dyskinesia, Tourette’s syndrome, and Meige’s syndrome have shown increased blink rates, while those with Parkinson’s disease and PSP exhibit decreased blink rates [95, 97–99].

### Blepharospasm and apraxia of eyelid opening

#### What are blepharospasm and apraxia of eyelid opening?

Spontaneous, erratic, and strong contractions of the orbital, preseptal, and pretarsal segments of the orbicularis oculi are characteristic of blepharospasm (BSP) [94]. They are typical dystonic indicators of PSP, often related to visual disabilities including functional blindness [100, 101]. Idiopathic BSP and secondary BSP represent the two major forms. Considering the cooccurrence of BSP and other uncharacteristic physical findings including parkinsonism and dementia may aid physicians to make a conclusive diagnosis of PSP [102]. Limb and axial dystonia, oromandibular dystonia, torticollis, and dysfluency (dystonia of the articulation muscle) represent additional focal dystonias other than BSP in PSP [101, 103]. Botulinum toxin usually results in good responses in patients [102]. BSP can be differentiated from apraxia of eyelid opening (ALO) on the basis of dissimilar responses after botulinum neurotoxin treatment [104].

Usually resulting from a lack of supranuclear control of the levator palpebrae and orbicularis oculi muscles, ALO is marked by the incapacity to open one’s eyelid [105, 106]. The majority of patients with this condition stemming from a lack of eyelid elevation control tend to contract their frontalis muscles more strongly to open their eyes instead of using their orbicularis oculi muscles, causing a noticeable forehead wrinkle. In PSP, ALO is a fairly common form of eyelid disorder [37, 94]. If botulinum neurotoxin cannot improve BSP notwithstanding the construction of eyelid weakness, ALO is a significant factor [104]. Due to the selective contraction of the pretarsal section of the orbicularis oculi potentially being responsible for eyelid closure, apraxia is often mistaken [104].

#### How can BSP and ALO be provoked?

A physician, typically a neurologist or ophthalmologist, is responsible for making a BSP diagnosis during a clinical assessment. While physical and neurological check-ups help offer a diagnosis, it is usually compulsory for the physician to verify the information given by the patient. By prompting a patient to move his/her face into both spontaneous and trained positions based on the physician’s directives, BSP can be recognized. By asking patients to shut their eyes forcefully, physicians can assess the bilateral contractions of the orbicularis oculi muscles. After patients open their eyes again, BSP may be observed as a continuing contraction of the orbicularis oculi muscles, related to a sinking of the eyebrows under the superior orbital rim (*Charcot’s sign*) [107].

By asking patients to close their eyes, ALO can be recognized. Patients are asked to open their eyes again quickly after closing them. Any incapacity to lift the eyelid without major orbicularis oculi muscle contraction may also be classified as ALO [105, 108]. While a patient is starting to open their eyes, excessive frontalis muscle contraction is typically observed as a forehead wrinkle.

#### Clinical effects of BSP and ALO

More prevalent in atypical parkinsonism than PD, BSP is a significant characteristic eye sign in PSP [37, 102, 109]. In varying levels of acuteness, nine articles have described BSP in patients with PSP as follows [37, 59, 61, 94, 100–102, 109, 110].

BSP in PSP could be serious and cause acute visual disability [94, 100]. The intensity and occurrence of BSP may also increase under certain conditions, as found in these studies. These conditions include exposure to bright light, watching television, reading, stress, and driving. Prior to the emergence of typical blepharospasm, more than one-third of patients with PSP suffer from excessive eye blinking [100]. Basic visual tasks could improve BSP, while direct commands commonly have no effect, as found by past research [94]. BSP in PSP leads to deteriorating changes involving the upper brainstem, without a true clinicopathological link to particular sites, based on a clinicopathological study [59]. As the result of dopaminergic medication, however, dystonia and BSP have been recounted in PSP [101, 109]. Levodopa administration was found to be linked to roughly 24% of cases with BSP in patients with PSP [101]. Further, previous research has found that BSP incidences occur late in the disease as well as at the same time with ALO [37, 61, 94, 102, 109]. Thus, the overlap of BSP and ALO could be an indication of PSP [109]. Similarly to BSP stemming from other causes, BSP in PSP typically responds to botulinum toxin injections [37]. Nonetheless, botulinum toxin injections may not achieve a full response in patients with PSP with isolated BSP when considering who had both BSP and ALO conditions [110].

Concerning research on ALO in patients with PSP, 14 articles have been identified as follows [18, 37, 58, 59, 62, 94, 105, 106, 108, 111–115].

ALO initially gained focus in 1965 as a non-paralytic abnormality characterized by difficulty in performing eyelid elevation [105]. An interruption in supranuclear control of the levator palpebrae and orbicularis oculi muscles may be a main contributor of ALO, causing difficulty in opening the eyes [106]. The benchmarks of ALO can be described as follows: (a) temporary incapability to raise the eyelids; (b) lack of progressive orbicularis oculi muscle contractions; (c) vigorous frontalis muscle contraction combined with incapacity to raise the eyelids; and (d) lack of oculomotor or ocular sympathetic nerve dysfunction as well as lack of ocular myopathy [94]. The most frequent eye sign in patients with PSP who fulfil the criteria of NINDS-SPSP for identification of PSP continues to be ALO, as long established in pathologically established cases of PSP [18, 58, 59, 62, 111]. Although ALO is a belated occurrence in PSP, in one out of six PSP cases it may represent the initial clinical presentation of PSP, at an average of 3.5 years after disease onset [62]. Further, ALO resulting from dopaminergic medications has been revealed in patients with parkinsonism [114, 115]. In a group of 32 patients with ALO of assorted aetiologies who reacted well to botulinum toxin injections, seven were patients with PSP [108]. This study found that ALO may be caused by focal eyelid dystonia instead of true apraxia, because of positive electrophysiologic findings with remote spasms of the pretarsal segment absent but complete contribution of the orbicularis oculi [108]. An incapability to constrain the voluntary contraction of the pretarsal portion of the orbicularis oculi muscle without clinically ongoing contraction of the orbicularis oculi muscle was detected in three patients with eyelid opening apraxia, termed ‘motor persistence of orbicularis oculi muscle’, comparable to the findings in a case study involving three patients with PSP. Subsequent to botulinum toxin injections into their eyelids, all three patients recovered [112]. The hypothesized eyelid dystonia in a report of two patients with PSP with ALO using a suggested injection site was restricted to the joint of the preseptal and pretarsal part of the orbicularis oculi muscles, as verified by the effective treatment response [113]. However, the benefits of botulinum toxin injections were identified in patients with a combination of ALO and BSP only, as proposed by a contrary study [37].

### How can a bedside ocular examination be performed to identify PSP?

The three necessary actions for examination of the eye (see Additional file 5) are described in detail in this section. It should be noted that the existence of any ocular abnormalities could indicate PSP when found in those with parkinsonism or dementia. For the distinction between PSP and other parkinsonian disorders, such abnormalities can be important in aiding the physician’s diagnosis. Bedside ocular examinations are typically carried out in the following three steps:

- ***Step 1***: By asking the patient to keep their gaze fixated on a stationary object for a period of 10 to 30 s, ocular fixation is established. Initial saccades move the fovea away from the intended position of fixation.
- ***Step 2***: The patient is prompted to move his/her eyes quickly to a moving target, which enables the establishment of ocular movement. It can then be assessed whether or not the patient is suitable for a performance of full eye excursion in the vertical as well as horizontal direction, while the head remains in a stationary position. The distance and rate of eye motion in both planes are observed in order to determine which plane is more flawed. To evaluate the VOR and decide whether to elicit the full vertical excursion with the eyes fixed on a target, the rotation of the head is assessed.
- ***Step 3***: Eyelid abnormalities are evaluated by asking the patient to close his/her eyes. After the eyes are closed, the patients will be asked to reopen their eyes again, to gauge the speed at which opening occurs. This enables the observer to check for orbicularis oculi muscle contractions.

**Additional file 5:** Video demonstrates the three necessary actions for examination of the eye. (MP4 9197 kb)


## Conclusions

When occurring in a patient with parkinsonism or dementia, distinct ocular abnormalities, which may include square wave jerks, slow vertical saccades, vertical supranuclear gaze palsy, ‘round the houses’ sign, decreased blink rates, blepharospasm, and apraxia of eyelid opening, could indicate PSP. For the discrimination of PSP from other parkinsonian disorders, such abnormalities can be important in aiding the physician’s diagnosis. The authors thus urge all concerned physicians to check for any abnormalities with the naked eye in patients with parkinsonism. This method has advantages, including ease of application, reduced time-consumption, and requirement of minimal resources. It will also help physicians to conduct efficient diagnoses since many patients with PSP could initially present with ocular symptoms in busy outpatient clinics.

## Additional files

Additional file 1: The various types of ocular abnormalities in PSP from systematic review. (DOCX 15 kb)

Additional file 2: Searching flow of systematic review. (PDF 156 kb)

Additional file 4: Video legend of the common seven eye signs of patients with progressive supranuclear palsy. (DOCX 15 kb)

## Data Availability

Not applicable.

## Abbreviations

ALO: Apraxia of eyelid opening
BSP: Blepharospasm
CBD: Corticobasal degeneration
CNS: Central nervous system
LPS: Levator palpebrae superioris
MLF: Medial longitudinal fasciculus
MSA: Multiple system atrophy
NPC: Niemann-Pick disease type C
PD: Parkinson’s disease
PPRF: Paramedian pontine reticular formation
PSP: Progressive supranuclear palsy
R2BRRC: R2 blink reflex recovery cycle
riMLF: Rostral interstitial nucleus of the medial longitudinal fasciculus
SWJs: Square wave jerks
VSP: Vertical supranuclear palsy
