# Supplementary material for: How to spot ocular abnormalities in progressive supranuclear palsy? A practical review
Source: Transl Neurodegener. 2019 Jul 10;8:20. doi: 10.1186/s40035-019-0160-1 (PMC6617936; doi:10.1186/s40035-019-0160-1)
Supplement: Supplementary file 3 — The various types of ocular abnormalities in PSP from systematic review. (DOCX 15 kb) [file 40035_2019_160_MOESM1_ESM.docx]

Additional data1: The various types of ocular abnormalities in PSP from systematic review

| Eye examination | Clinical findings | Eye examination | Clinical findings |
| --- | --- | --- | --- |
| Visual complaints | - Blurred vision | Ocular fixation | - Square wave jerks |
|  | - Eye pain or irritation |  | - Saccadic intrusions |
|  | - Diplopia | Pupillary function | - Decreased pupil diameter |
|  | - Photophobia | Eyelids | - Decreased eye blink |
| Eye movement | - Slow saccades |  | - Blepharospasm |
|  | - Hypometric saccades |  | - Apraxia of eyelid opening |
|  | - Vertical gaze palsy |  | - Apraxia of eyelid closure |
|  | - Lateral gaze palsy |  | - Lid retraction |
|  | - Abnormal smooth pursuit | Nystagmus | - Impaired optokinetic nystagmus |
|  | - “Round the Houses” sign | Miscellaneous | - Internuclear ophthalmoplegia |
|  | - Complete ophthalmoplegia |  | - Ocular flutter |
| VOR | - Impaired capacity to cancel gain |  | - Exodeviation |
