# Supplementary figures and images for: How to spot ocular abnormalities in progressive supranuclear palsy? A practical review
Source: Transl Neurodegener. 2019 Jul 10;8:20. doi: 10.1186/s40035-019-0160-1 (PMC6617936; doi:10.1186/s40035-019-0160-1)

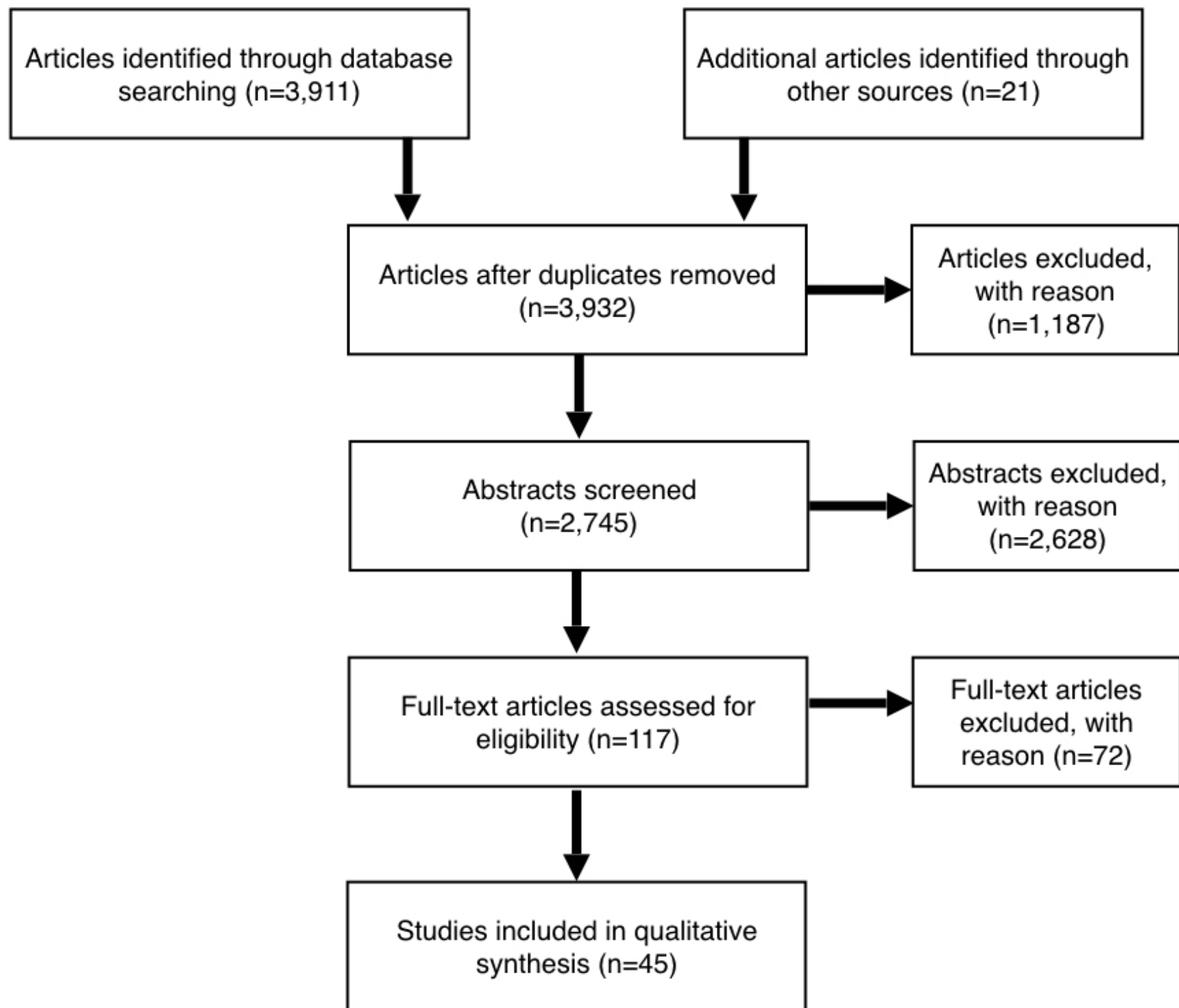

Supplement: Supplementary file 4 — Searching flow of systematic review. (PDF 156 kb) [file 40035_2019_160_MOESM2_ESM.pdf]
