# Supplementary material for: How to spot ocular abnormalities in progressive supranuclear palsy? A practical review
Source: Transl Neurodegener. 2019 Jul 10;8:20. doi: 10.1186/s40035-019-0160-1 (PMC6617936; doi:10.1186/s40035-019-0160-1)
Supplement: Supplementary file 5 — Video legend of the common seven eye signs of patients with progressive supranuclear palsy. (DOCX 15 kb) [file 40035_2019_160_MOESM4_ESM.docx]

**Additional data 4**

Video legend of the seven common eye signs for patients with progressive supranuclear palsy ordered as follows: square wave jerks, slow saccades, ‘Round the Houses’ sign, vertical supranuclear gaze palsy, decreased blink rate, blepharospasm, and apraxia of eyelid opening.

***Video segment 1: Square wave jerks***

Video of a PSP patient who was diagnosed with PSP for three years without the establishment of vertical supranuclear gaze palsy showed abnormal eye fixation while focusing on a stationary target. Involuntary, horizontal, saccadic intrusions that interrupted fixation or square wave jerks occurred*.*

***Video segment 2: Slow vertical saccades***

Video of a PSP patient who was diagnosed with PSP for three years showed abnormal eye movement while performing saccades. Slowness of vertical saccades disproportionate to horizontal saccades was shown. He could undergo performance of fully vertical excursion without evidence of vertical supranuclear gaze palsy or ophthalmoparesis.

***Video segment 3: ‘Round the Houses’ sign***

Video of a PSP patient who was diagnosed with PSP for three years showed abnormal eye movement while performing saccades. Slowness of vertical saccades disproportionate to horizontal saccades was shown without evidence of ophthalmoparesis. While performing vertical saccades, the patient displayed an inability to move her eyes upward or downward in straight lines along a midline, instead showing the curved course of oblique saccades, appearing as a circle called the “Round the Houses” sign or “Roundabout” sign.

***Video segment 4: Vertical supranuclear gaze palsy***

Video of a PSP patient who was diagnosed with PSP for five years showed abnormal movement while performing saccades. Slowness and hypometria of the vertical saccades disproportionate to the horizontal saccades were shown without evidence of total ophthalmoparesis. His vertically hypometric gaze with reversing gain after performing the vestibulo-ocular reflex (VOR) supported the etiologically of vertical supranuclear gaze palsy.

***Video segment 5: Decreased blink rate***

Video of a PSP patient who was diagnosed with PSP for seven years showed a decreased blink rate. By counting how many times per minute a person blinks during a 5-minute conversation, decreased eye blink rate can be identified. When at least 50% closure of the eyes occurs with a quick and subsequent reopening of the eyes, blinks are deemed to be full blinks.

***Video segment 6: Blepharospasm***

Video of a PSP patient who was diagnosed with PSP for seven years showed forceful contraction of her eyelids, which confirmed the diagnosis of blepharospasm. Forceful contraction of the orbicularis oculi muscles without significant frontalis muscle contraction was observed, leading to the patient being unable to open her eyes, which caused significant visual disability. She also had lip’s contraction and mild cervical dystonia.

***Video segment 7: apraxia of eyelid opening***

Video of a PSP patient who was diagnosed with PSP for seven years showed persistent closing of the eyes. Further, he could not initiate his eyelids to open after given a command. During periods of inability to raise the eyelids, forceful contraction of the orbicularis oculi was not observed, whereas, contraction of the frontalis muscle was observed and presented as a wrinkle on his forehead. Once, the patient was asked to open his eyes, but he had difficulty doing so. As a result, he attempted to use both hands to touch and open his eyelids.
